# Progress and Challenges of Ultrasonic Testing for Stress in Remanufacturing Laser Cladding Coating

**DOI:** 10.3390/ma11020293

**Published:** 2018-02-13

**Authors:** Xiao-Ling Yan, Shi-Yun Dong, Bin-Shi Xu, Yong Cao

**Affiliations:** 1 College of Material and Mechanical Engineering, Beijing Technology and Business University, Beijing 102488, China; 2 National Key Laboratory for Remanufacturing, Academy of Army Armored Forces, Beijing 100072, China; dongsy73@gmail.com (S.-Y.D.); wydyxl1974@163.com (B.-S.X.); wwlwyd2002@163.com (Y.C.)

**Keywords:** remanufacture, laser cladding forming, stress, ultrasonic testing, complex factors coupling

## Abstract

Stress in laser cladding coating is an important factor affecting the safe operation of remanufacturing components. Ultrasonic testing has become a popular approach in the nondestructive evaluation of stress, because it has the advantages of safety, nondestructiveness, and online detection. This paper provides a review of ultrasonic testing for stress in remanufacturing laser cladding coating. It summarizes the recent research outcomes on ultrasonic testing for stress, and analyzes the mechanism of ultrasonic testing for stress. Remanufacturing laser cladding coating shows typical anisotropic behaviors. The ultrasonic testing signal in laser cladding coating is influenced by many complex factors, such as microstructure, defect, temperature, and surface roughness, among others. At present, ultrasonic testing for stress in laser cladding coating can only be done roughly. This paper discusses the active mechanism of micro/macro factors in the reliability of stress measurement, as well as the impact of stress measurement on the quality and safety of remanufacturing components. Based on the discussion, this paper proposes strategies to nondestructively, rapidly, and accurately measure stress in remanufacturing laser cladding coating.

## 1. Introduction

Due to its technological advantages, laser cladding [1,2] has become an important method of green remanufacturing [3] for old products. In the process of laser cladding, a high-energy laser beam is used as a moving heat source.

Rapid heating, melting, and cooling provide extreme non-equilibrium conditions that cannot be achieved by conventional methods, so that forming parts gain excellent comprehensive properities. Laser cladding has become an advanced technology in remanufacturing engineering [4]. Because of the uneven heating in the process of laser cladding, as well as the difference of the thermal expansion coefficient among different compositions in laser cladding coating, the stress distribution in laser cladding coating is complicated [5,6]. Stress [7,8] has become a key factor that affects the service performance and service life of remanufacturing mechanical parts. With the development of laser cladding remanufacturing technology, the stress evaluation of remanufacturing coating becomes more and more important. The key issues in this field have shifted from the study of the apparent problems of quality and performance of remanufacturing products to the deep-seated problems of high quality and reliability assurance. At present, the stress testing methods [9] can be divided into two categories: destructive methods (the small blind hole method [10], the stripping layer method [11], and the ring core method [12]) and nondestructive methods (the ray diffraction method [13], the magnetic memory method [14], the optical method [15], and the ultrasonic method [16,17,18]). Although these methods can determine stress measurements, there are some problems which cannot be ignored; for example, the destructive methods destroy the integrity of the component structure and can only realize the sampling testing, while the X-ray diffraction method is harmful to the health of the operator. The optical method has high requirements for the detection environment and cannot be detected online. The magnetic memory method has limitations, since only ferromagnetic materials can be detected. The ultrasonic method [19,20,21] has many advantages, such as a wide detection range, safety, non-destructive detection, and online detection. Therefore, the ultrasonic method has become a popular research direction in non-destructive stress testing.

To ensure the service reliability of laser cladding remanufacturing components, the primary challenge is to evaluate and control the residual stress and initial defects of laser cladding coating. The evaluation of residual stress and initial defects can provide guidance for the optimization of the laser cladding process [22,23], so as to improve the service safety of remanufacturing components. On this basis, the changing lifetime of laser cladding remanufacturing components is discussed, which provides insight into the multi-life cycles of components.

At present, the theory and technology of the non-destructive evaluation of defects in laser cladding coating are mature [24,25], but the non-destructive evaluation of stress in laser cladding coating is still at the “experience” and “rough” levels; the related theory and experimental research have not yet been perfected. In view of the advantages of the ultrasonic method in nondestructive stress testing, this paper introduces the theory and method of ultrasonic testing for stress in laser cladding coating, the interaction mechanism between anisotropic microstructures, and ultrasonic testing signals.

## 2. Basic Theory of Ultrasonic Testing for Stress

The method for stress testing by ultrasonic wave is based on acoustoelasticity theory [26] and nonlinear ultrasonic theory [27]. The application of acoustoelasticity theory is based on the establishment of a series of assumptions. These assumptions include objects possessing the characteristics of continuity and uniformity; objects being hyperelastic; microvariations of ultrasonic waves being superimposed on the finite deformation of objects; the deformation process being isentropic. Nonlinear ultrasonic testing for stress is based on the nonlinear characteristics of ultrasonic waves propagating in a solid medium (under stress). The appearance of nonlinear effects in elastic wave propagation is one of the most reliable and sensitive indicators of the onset of material damage [28]. 

### 2.1. Acoustoelasticity Theory 

In 1953, Hughes [29] and Kelly [30] proposed the early expressions of stress and ultrasonic velocity in isotropic solids based on the finite deformation theory, which laid the foundation of acoustoelasticity theory. Since the 1960s, the acoustoelasticity effect has been continuously improved by R.A. Toupin, B. Bernstein [31,32], R.N. Thurston, et al. [33].

In summary, the existing expressions for acoustoelasticity equations of isotropic solids are as follows [29,30]:

(1) The direction of longitudinal wave propagation is parallel to the stress direction:
  (1) $$\rho v_{111}^{2}=\lambda +2u+\frac{\sigma }{3K_{0}}[2l+\lambda +\frac{\lambda +\mu }{\mu }(4m+4\lambda +10\mu )]$$
(2) The direction of longitudinal wave propagation is perpendicular to the stress direction:
  (2) $$\rho v_{113}^{2}=\lambda +2u+\frac{\sigma }{3K_{0}}[2l-\frac{2\lambda }{\mu }(m+\lambda +2\mu )]$$
(3) The direction of transverse wave propagation and the direction of polarization are parallel and perpendicular to the stress direction, respectively:
  (3) $$\rho v_{131}^{2}=\mu +\frac{\sigma }{3K_{0}}(m+\frac{\lambda n}{4\mu }+4\lambda +4\mu )$$
(4) The direction of transverse wave propagation and the direction of polarization are perpendicular to the stress direction:
  (4) $$\rho v_{132}^{2}=\mu +\frac{\sigma }{3K_{0}}(m-\frac{\lambda +\mu }{2\mu }n-2\lambda )$$
(5) The direction of transverse wave propagation and the direction of polarization are perpendicular and parallel to the stress direction, respectively:
  (5) $$\rho v_{133}^{2}=\mu +\frac{\sigma }{3K_{0}}(m+\frac{\lambda n}{4\mu }+\lambda +2\mu )$$
(6) Longitudinal wave under static pressure:
  (6) $$\rho v_{111}^{2}=\lambda +2\mu -\frac{\sigma }{3K_{0}}(6l+4m+7\lambda +10\mu )$$
(7) Transverse wave under static pressure:
  (7) $$\rho v_{131}^{2}=\lambda -\frac{\sigma }{3K_{0}}(3m-\frac{1}{2}n+3\lambda +6\mu )$$

where *v*_ijk_ is the velocity of the ultrasonic wave, the first subscript *i* is the propagation direction of the ultrasonic wave, the second subscript *j* is the polarization direction of the ultrasonic wave, the third subscript *k* is the direction of uniaxial stress, ρ is the density of the isotropic solid, σ is the stress in the isotropic solid, *K*_0_ is the bulk modulus, λ,u are the second order elastic constants, m,n,l are the third order elastic constants.

Remanufacturing laser cladding coating is usually anisotropic; in the process of laser cladding, the material undergoes elastic-plastic deformation. Acoustoelasticity theory, which is perfectly consistent with the elastic-plastic deformation, needs to be developed further. Some universities and scientific research institutions in the United States, Japan, and Britain have carried out a series of related studies and achieved some significant results since the 1980s. In 1981, Johson [34,35] deduced the acoustic elastic formula under elastoplastic conditions based on the elastic-plastic continuum model of Green. Elastic strain, plastic strain, principal stretch ratio, and strength hardening parameters are included in the formula; these complex parameters can be determined by elastoplastic experimentation, so it is difficult for the formula to be popularized and applied in practice. In the same year, Okada [36] derived the acoustic elastic formula for weakly orthotropic materials under the assumption of the nonlinear elastic constitutive relation of anisotropic materials. In 1985, Pao [37] deduced the acoustic elastic formula in the orthotropic medium with initial stress. The above research results are beneficial to the acoustoelasticity theory in anisotropic materials under elastic-plastic deformation, but most of these researches remain at the theoretical level and are far from practical application.

### 2.2. Nonlinear Acoustoelasticity Theory

As early as 1755, Euler proposed the concept of nonlinear acoustics, while Lagrange, Strokes, and Rayleigh studied nonlinear acoustic theory [38]. In the 1960s, researchers began to study nonlinear acoustic phenomena in solids. In 1963, the phenomenon of harmonic propagation in aluminum was observed by Hikata in the metals laboratory at Brown University [39]. 

The laser cladding coating with residual stress has nonlinear characteristics of solids; when the ultrasonic wave propagates, the nonlinear characteristics of laser cladding coating can be characterized by the nonlinear phenomena of ultrasonic propagation. When a one-dimensional longitudinal wave propagates through a nonlinear medium, in the case of small strain, the equation of the longitudinal wave’s motion can be written as [40,41]:

(8) $$\frac{\rho^{2}}{E^{2}}\frac{\partial^{2}u}{\partial t^{2}}=\frac{\partial^{2}u}{\partial x^{2}}+\beta \frac{\partial u}{\partial x}\frac{\partial^{2}u}{\partial x^{2}}+\delta {\left(\frac{\partial u}{\partial x}\right)}^{2}\frac{\partial^{2}u}{\partial x^{2}}$$

where ρ is the density of medium, *E* is elastic modulus, *u* is the displacement in the *x* direction, and β,δ are called the second and third order nonlinear coefficients, respectively. They are related to the second, third, and fourth order elastic constants of the material.

Since Equation (8) has no general analytic solution, the perturbation method is adopted by domestic and overseas scholars to obtain its approximate solution. The perturbation method is mainly intended to expand the required parameter as a power series; here, *u*(*x,t*) is expanded by the power of *x*. Finally, the power series expansion results are simplified and consolidated according to the same power of *x*, thus finding the solution of Equation (8) [40,41]:

(9) $$u(x,t)=A_{1}\cos(kx-\omega t)-\frac{\beta }{8}k^{2}A_{1}^{2}x\cos2(kx-\omega t)+\frac{\delta }{24}k^{3}A_{1}^{3}x[\cos3(kx-\omega t)+3\cos(kx-\omega t)]$$

where ω is circular frequency, *k* is wave number, *A*_1_ is the amplitude of the fundamental wave, *A*_2_ = $\frac{\beta }{8}k^{2}A_{1}^{2}x$ is the amplitude of the second harmonic, and *A*_3_ = $\frac{\delta }{24}k^{3}A_{1}^{3}x$ is the amplitude of the third harmonic. Therefore, the expressions for the second and third order nonlinear coefficients are as follows:

(10) $$\beta =\frac{8A_{2}}{k^{2}A_{1}^{2}x}$$

(11) $$\delta =\frac{24A_{3}}{k^{3}A_{1}^{3}x}$$

It can be seen from Equations (10) and (11) that the amplitude of the second harmonic (*A*_2_) and the amplitude of the third harmonic (*A*_3_) depend on the nonlinear parameter β,δ, respectively. The two parameters indicate the characteristics of material related to stress. Therefore, if the β,δ can be measured, the stress state of the material can be estimated. Due to the symmetry of the third order elastic constants in laser cladding coating, the shear wave in the surface wave does not produce harmonic components [42,43], so the description of the nonlinear coefficients for the longitudinal wave is also applicable to the surface wave.

## 3. Ultrasonic Testing for Stress in Laser Cladding Coating

Ultrasonic testing has become a popular direction in the nondestructive evaluation of stress, because it has the advantages of safety, nondestructiveness, and online detection. Remanufacturing laser cladding coating shows typical anisotropic behaviors, while the ultrasonic testing signal in laser cladding coating is influenced by many complex factors [16]. At present, the nondestructive evaluation of stress in laser cladding coating can only be done roughly.

### 3.1. Ultrasonic Testing Methods for Stress

The main methods for testing stress based on acoustoelasticity theory include the use of the relationship between ultrasonic velocity and stress [44]; ultrasonic attenuation degree and stress [45]; the incident angle of Rayleigh wave and stress [46]; echo power spectrum and stress [47]; and the interaction of acoustic beams and stress [48]. The excitation waveforms used to measure stress in laser cladding coating include Rayleigh waves [16], critical refraction longitudinal waves [49], or two wave combinations [50].

**Figure 1:**
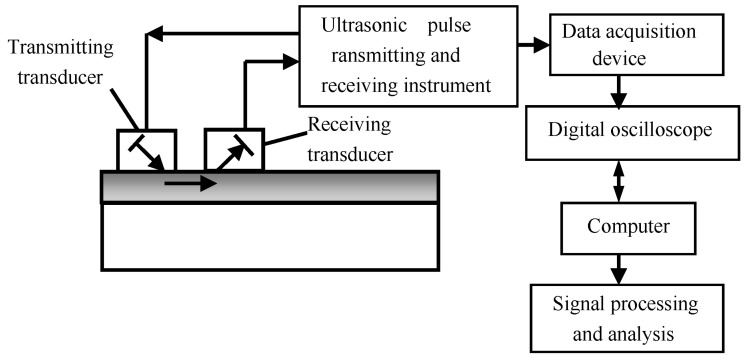
A classical ultrasonic testing system for stress in laser cladding coating [51]. “Reproduced with permission from publisher by © Springer.” (2015).

The relationship between ultrasonic velocity and stress is the focus of current research [16,49,50]. A classical stress measurement system based on the relationship between Rayleigh wave velocity and stress is shown in Figure 1 [51]. It mainly consists of a Panametrics-NDT 5800PR ultrasonic pulse transmitting (Panametrics-NDT, Waltham, MA, USA) and receiving instrument, a TDS5000B oscilloscope (highest sampling frequency is 2.5 GHz, Tektronix, Beaverton, WA, USA), and Rayleigh wave transducers (SIUI, Shantou, China), with a frequency of 5 MHz (a transmitting transducer and a receiving transducer). In the experiment, in order to ensure the coupling between the tranducer and the sample is stable, the contact between them is elastic. Additionally, a simple device is used to fix the Rayleigh wave transducer to the detected area to collect data (the distance between the tranducers is 20 mm). Because the Rayleigh wave velocity is inconvenient to measure, and the propagation time of Rayleigh waves can be measured directly, the velocity is converted into the change rate of propagation time within a certain distance.

Both domestically and internationally, there are still few researches on stress testing based on nonlinear ultrasonic theory. In 2009, Chaki et al. [52] analyzed the stress value of prestressed steel strands by using the nonlinear ultrasonic guided wave technique, and discussed the sensitivity of different modes of guided wave to stress. In 2010, Liu et al. [53] used nonlinear Rayleigh waves to test the residual stress in the aluminum alloy plate produced by shot peening. It was found that the stress values were in one-to-one correspondence with the nonlinear coefficients. The above researches on ultrasonic testing for stress in heterogeneous materials have strong novelty and reference significance. However, it must also be acknowledged that most of the researches remain at the level of experimental observation, and lack deep theoretical analysis.

### 3.2. The Influence Mechanism of Micro Factors on Ultrasonic Testing for Stress

**Figure 2:**
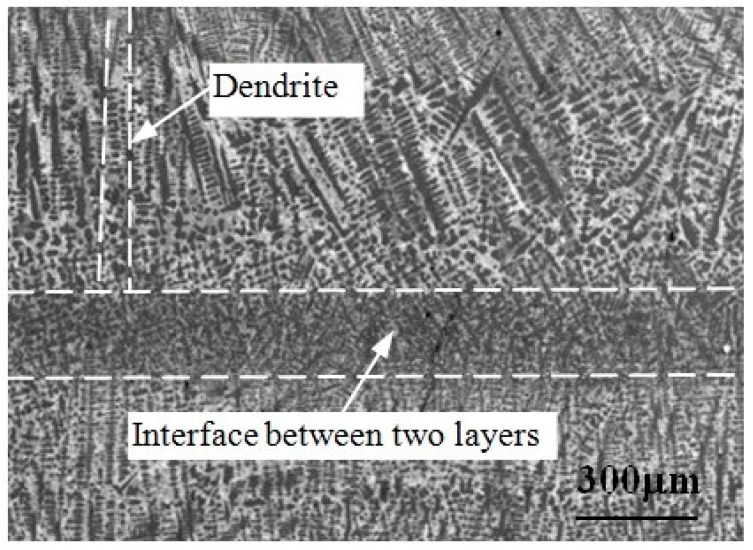
SEM micrograph of Fe314 laser cladding coating [51]. “Reproduced with permission from publisher by © Springer.” (2015).

**Figure 3:**
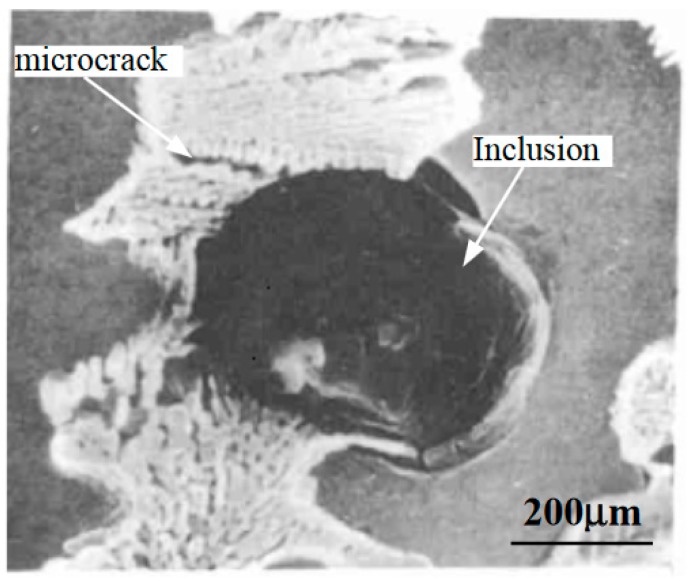
SEM micrograph of Fe_55_Cr_20_Ni_10_B_2_Si_2_ laser cladding coating.

The microstructure of remanufacturing laser cladding coating is usually anisotropic. Figure 2 [51] shows the SEM micrograph of Fe314 laser cladding coating. As can be seen in Figure 2, the interior is obviously dendritic. Because the laser cladding sample was prepared by means of multilayer and multipass lap cladding, there is obvious interface between two layers in the laser cladding coating. The growth direction of dendrite in a single layer is basically identical, which is approximately perpendicular to the interface between two layers. The continuity of dendrite growth in the adjacent laser cladding layer is interrupted by the interface between two layers, and the growth direction is slightly different. The adjacent cladding layers are bonded together by metallurgical bonding, which ensures the continuity of dendrite growth in the inner layer and the strength of interlayer bonding. Figure 3 shows the SEM micrograph of Fe_55_Cr_20_Ni_10_B_2_Si_2_ laser cladding coating. Inclusions and microcracks at their boundaries can be seen in Figure 3. 

Textures, inclusions, and defects in the laser cladding coating are called micro factors. The question is how these micro factors affect the stress testing results based on ultrasonic waves. Experimental studies have been carried out by related universities and research institutions. In 1983, King [54] used the oblique incidence horizontal polarization shear wave (*SH* wave) to achieve the measurement of plane stress state under weak orthogonality conditions. This method effectively separated texture effects and stress effects. It is assumed that the principal stress coincides with the material symmetry axis, while the acoustoelasticity equation in the plane stress state is as follows:

(12) $$\frac{SH_{23}-SH_{13}}{SH_{0}}=\frac{c_{55}-c_{44}}{c_{44}}\cos^{2}\theta +\alpha (\theta )(T_{22}-T_{11})$$

where *SH_ij_* is the velocity of *SH* wave propagating in the surface *ij*, *SH*_0_ is the average velocity of two kinds of *SH* wave, *c*_44_, *c*_55_ are the elastic constants of the material, α(θ) is the elastic constant at different angles, and *T*_22_, *T*_11_ are the principal stress.

In 1984, Thompson [55] adopted plane *SH* waves propagating in a vertical direction to separate texture effects and stress effects. The expression is presented as:

(13) $$\rho (v_{ij}^{2}-v_{ji}^{2})=\sigma_{ii}-\sigma_{jj}$$

where $v_{ij},v_{ji}$ is the velocity of *SH* waves propagating in the surface *ij*, and $\sigma_{ii},\sigma_{jj}$ are the principal stress.

In 1984, Allen and Sayeres [50] used the method of combining focused P-wave and S-wave birefringence to separate tissue effects. The method was validated by measuring the residual stress at the crack tip. In 1992, Rokhlin [56] proposed GAO technology(using two kinds of transverse waves and one kind of longitudinal wave); as a result, the relation between the polarization angle of the transverse wave and stress was established. In 2002, the residual stress in butt-welded plates was measured by using the critical refraction longitudinal wave in the French Mechanical Industry Technology Center [57]. The effect of microstructure in the heat–affected zone and weld–zone on the test results was considered, and the results were in good agreement with the small bore method. In 2015, Rayleigh waves were used [51] to test the stress in laser cladding coating, and analyzed the effect of the anisotropic microstructure on the testing results, in combination with the theory of elastic-plastic deformation. The expression proposed is:

(14) $$\left\{ \begin{array}{l} \frac{v_{1}-v_{1}^{0}}{v_{1}^{0}}=k_{1}\sigma_{1}+k_{2}\sigma_{2}+\alpha_{1}\\ \frac{v_{2}-v_{2}^{0}}{v_{2}^{0}}=k_{2}\sigma_{1}+k_{1}\sigma_{2}+\alpha_{2} \end{array}\right.$$

where $v_{i},v_{i}^{0}$ is the velocity of the Rayleigh wave propagating in laser cladding coating under the condition of stress and no stress respectively; *k*_1_, *k*_2_ is the acoustic elastic coefficient of laser cladding coating in two directions perpendicular to each other respectively; $\alpha_{1},\alpha_{2}$ is the tissue effect factor in two directions perpendicular to each other respectively.

**Figure 4:**
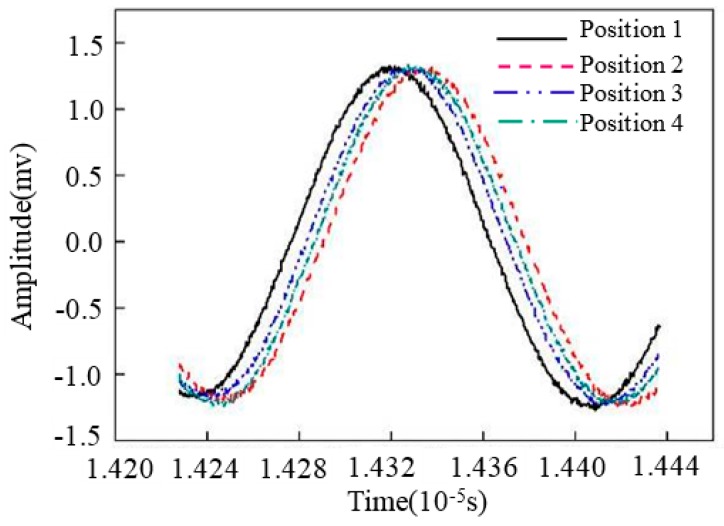
Rayleigh wave signals of laser cladding Fe314 alloy coating responding to the zero stress [51]. “Reproduced with permission from publisher by © Springer.” (2015).

**Figure 5:**
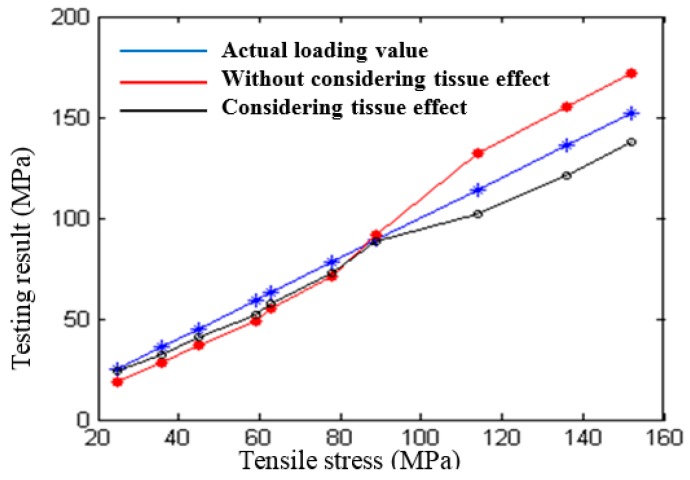
Stress testing results of Fe314 laser cladding coating [51]. “Reproduced with permission from publisher by © Springer.” (2015).

Figure 4 [51] shows the Rayleigh wave signals propagating in a fixed acoustic path according to different testing positions of Fe314 laser cladding coating (the stress is zero). Figure 5 [51] shows the results of stress testing before and after removal of the tissue effect. It can be seen that the method proposed by the author’s research group can effectively improve the reliability of the testing results. The above researches have greatly promoted the development of ultrasonic nondestructive testing for stress in anisotropic materials, and have strong reference significance. However, there is some blindness in exploring the method of separating tissue effects. The fundamental reason for this blindess is that the ultrasonic propagation theory is not combined with the elasto-plastic deformation theory, the influence mechanism of tissue effects on ultrasonic testing for stress in anisotropic materials is not analyzed in depth, and a convincing explanation is not given for the separation of tissue effects.

### 3.3. The Influence Mechanism of Macro Factors on Ultrasonic Testing for Stress

**Figure 6:**
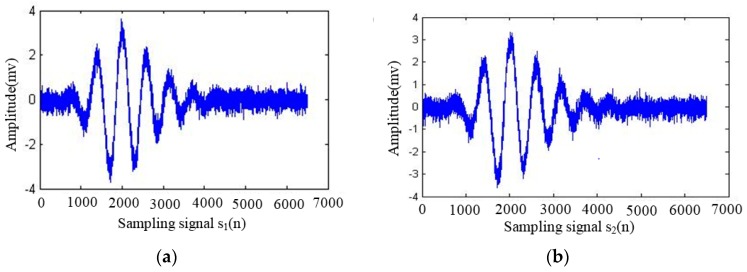
Time-domain diagram of the sampling signal [66]. (**a**) Under 34 MPa tensile stress; (**b**) under 230 MPa tensile stress. “Reproduced with permission from publisher by © Zhongguo Zhendong Gongcheng Xuehui.” (2013).

**Figure 7:**
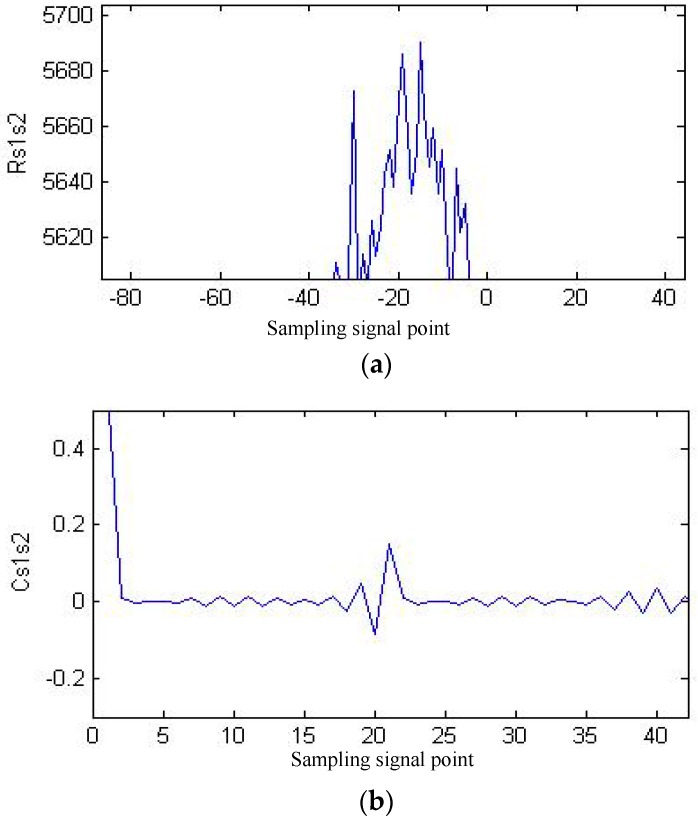
Contrast of acoustic time delay analysis results [66]. (**a**) Correlation analysis result; (**b**) complex cepstrum analysis result “Reproduced with permission from publisher by © Zhongguo Zhendong Gongcheng Xuehui.” (2013).

Temperature, probe coupling mode, coupling layer thickness, and surface roughness are called macro factors. These macro factors have various effects onstress testing results based on ultrasonic waves. The most influential methods currently include (1) exploring the influence of macro factors on stress testing results through tentative experiments, and proposing methods for correcting errors [58,59,60]; (2) improving the accuracy of testing characteristic parameters (such as ultrasonic signal propagation speed, amplitude, etc.) in ultrasonic testing for stress experiments [61,62,63]; and (3), exploring the methods for correcting errors through tentative experiments (once the testing object and experimental condition change, many experiments are needed to find the method to correct the error). Modern signal processing technology can improve the reliability and accuracy of stress testing results to a certain extent [64,65]. Figure 6a,b [66] are Rayleigh wave signals propagating in a fixed acoustic path in Fe314 laser cladding coating under 34 MPa and 230 MPa tensile stress, respectively. It can be seen in Figure 6 that the acquired Rayleigh wave signals contain a lot of noise. Evaluating the stress of laser cladding coating based on Rayleigh waves, the extraction of acoustic time delay is a key technology. Figure 7a [66] shows the acoustic time delay analysis result based on the correlation method. Figure 7b [66] is the acoustic time delay analysis result based on the complex cepstrum method proposed by the author’s research group. As can be seen in Figure 7, the signal-to-noise ratio of correlation analysis is low. At the same time, the time delay peak is very close to the surrounding interference signal, so it is not easy to locate accurately. The signal-to-noise ratio of complex cepstrum analysis is relatively high, while the time delay peak is very sharp, and it is convenient for accurate positioning (the time difference between two signals is only 20 sampling points (8 ns)).

To sum up, there are still many basic scientific problems unsolved in the field of ultrasonic nondestructive testing for stress in laser cladding coating. These problems are summarized as follows: (1) the interaction mechanism of anisotropic laser cladding coating and ultrasonic testing signals is not clear; (2) the effect of laser cladding coating and the substrate “combination” and “sound transmission” on the stress testing result remains to be studied; (3) the essential principle for separation of tissue effects in anisotropic materials is not given; and (4), the stress testing for anisotropic materials based on nonlinear ultrasonic testing lack a profound theoretical analysis. The preliminary research of the author’s research group (the research group on nondestructive testing for quality of remanufacturing parts) showed that some basic scientific problems in the field of ultrasonic stress testing can be solved by using numerical simulation and data mining technologies [51,66].

## 4. Strategies for Solving Related Problems

To solve the technical bottleneck problems in ultrasonic testing for stress in laser cladding coating, research should grasp the principal contradiction and reduce the complex problems. The factors that affect the reliability of ultrasonic testing for stress in laser cladding coating are classified into two categories: micro and macro. The influences of micro factors and macro factors can be stripped. First, by changing the laser cladding process, specimens with different microstructure and stress state are prepared. In the process of testing, the stability of macro factors (such as environment, instrument, and personnel) is maintained, and the influence mechanism of micro factors on testing signal is studied. Second, in view of the same laser cladding sample (to ensure the consistency of micro factors), the change law of stress testing results is studied when the macro factor changes. Then, the coupling effect of macro and micro factors is considered. The micro and macro factors related to stress testing are extracted by data mining, and the coupling effect of multiple factors in ultrasonic stress tesing are proved by orthogonal and uniform experiments. Finally, the multiple factors coupling analysis results are used as Support Vector Machine (SVM) input samples [67]. The prediction model of optimum testing methods, based on multidimensional analysis and multi-source information, is established to realize nondestructive, fast, and reliable testing for the stress in remanufacturing laser cladding coating.

### 4.1. Obtain Full-Effective Acoustic Field Information

Compared with the experimental research, numerical simulation is more flexible and convenient in model making, parameter selection and variation, and data processing of simulation results. Numerical simulation can highlight some details which are not easily observed in the experiment. The numerical simulation technology of ultrasonic testing with the objective of acoustic field analysis and flaw echo prediction has developed rapidly in recent years. Its applications include (1) scattering and echo prediction of different types of flaws [68,69,70]; (2) ultrasonic propagation and acoustic field analysis in isotropic and anisotropic materials [71,72,73]; (3) ultrasonic imaging simulation [74,75]. To the best of our knowledge, no other scholars have carried out ultrasonic nondestructive testing for stress in anisotropic materials by means of numerical simulation.

**Figure 8:**
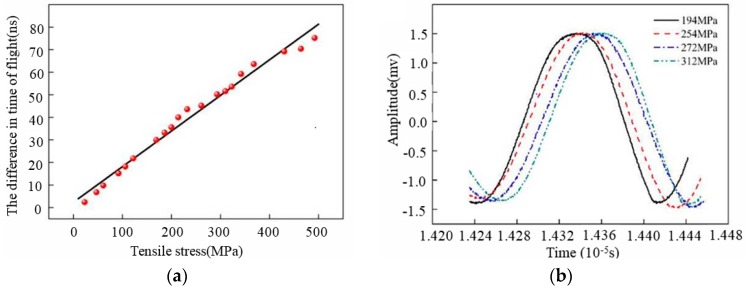
(**a**) Acoustic elastic curve of Rayleigh waves in aluminum alloy; (**b**) Rayleigh wave signals in aluminum alloy under different stress.

**Figure 9:**
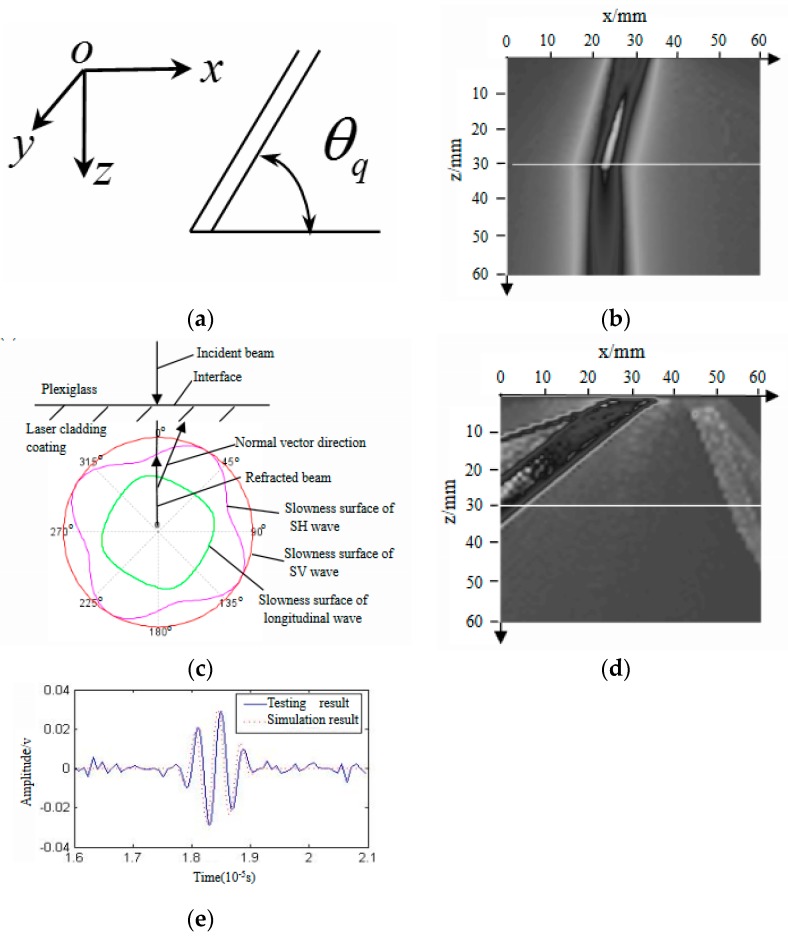
Numerical simulation results [77]. (**a**) The grain orientation angle $\theta_{q}$ and direction of the coordinate system; (**b**) The ultrasonic field radiated by a longitudinal wave straight probe in the Fe314 laser cladding remanufacturing specimen; (**c**) Schematic diagram of vertical incident longitudinal wave propagation in Fe314 laser cladding coating; (**d**) The ultrasonic field radiated by a transverse wave angle probe in the Fe314 laser cladding remanufacturing specimen; (**e**) Simulation and testing results of the flaw echo signal in the Fe314 laser cladding remanufacturing specimen. “Reproduced with permission from publisher by © Springer.” (2016).

The author’s research group, using ANSYS/LS-DYNA Finite Element Method (FEM) [76], obtained the acoustic elastic curve of Rayleigh waves in aluminum alloy (as shown in Figure 8a) through the implicit and explicit solution. The red point is the data collected during a static load test; the experimental results are in good agreement with the numerical simulation results. Figure 8b is the Rayleigh wave signals in aluminum alloy under different stress. Therefore, it is more convenient to obtain the intrinsic relationship between the velocity of ultrasonic signals and stress in material by using FEM. In addition, the influence of anisotropic laser cladding coating on the propagation behaviors of ultrasonic beams was analyzed by the author’s research group. The propagation behaviors of ultrasonic beam in the laser cladding Fe314 alloy coating were investigated with the help of Rayleigh integral, combined with the pencil method. Figure 9 [77] is part of the numerical simulation results. Figure 9a shows the grain orientation angle in laser cladding coating and the coordinate system direction. Figure 9b shows the numerical simulation results of ultrasonic field distribution in the xoz section of the Fe314 laser cladding remanufacturing specimen (the white line in Figure 9b is the joint surface of laser cladding coating and the substrate), based on the longitudinal wave vertical incidence and a grain orientation angle of 30°. The results show that the grain orientation can affect the propagation behavior of acoustic beams in the anisotropic laser cladding coating; when the grain orientation angle is 30°, the propagation direction of the vertical incidence longitudinal beam will change. Figure 9c shows the propagation principle of the vertical incident wave in Fe314 laser cladding coating. As can be seen in Figure 9c, when the grain orientation angle is 30°, the slowness surface of the longitudinal wave is not an ideal circle, so the direction of group velocity (the normal vector direction of the slowness surface) is inconsistent with that of phase velocity (the direction of a refraction longitudinal wave beam); thus, the longitudinal wave beam will change direction. As shown in Figure 9d, under the same conditions, the oblique incidence transverse beam not only changes direction but also splits into two shear beams with different propagation velocities. Figure 9e is the flaw echo in the Fe314 laser cladding remanufacturing specimen (the numerical simulation and test results are in good agreement). Thus, theoretical analysis, numerical simulation, and experimental detection can be verified mutually, while numerical simulation technology is more intuitive to analyze the propagation behaviors of ultrasonic signals in anisotropic materials. Using numerical simulation technology, the information collection and processing in the process of ultrasonic testing for stress are more transparent and detailed.

### 4.2. Technology Roadmap

**Figure 10:**
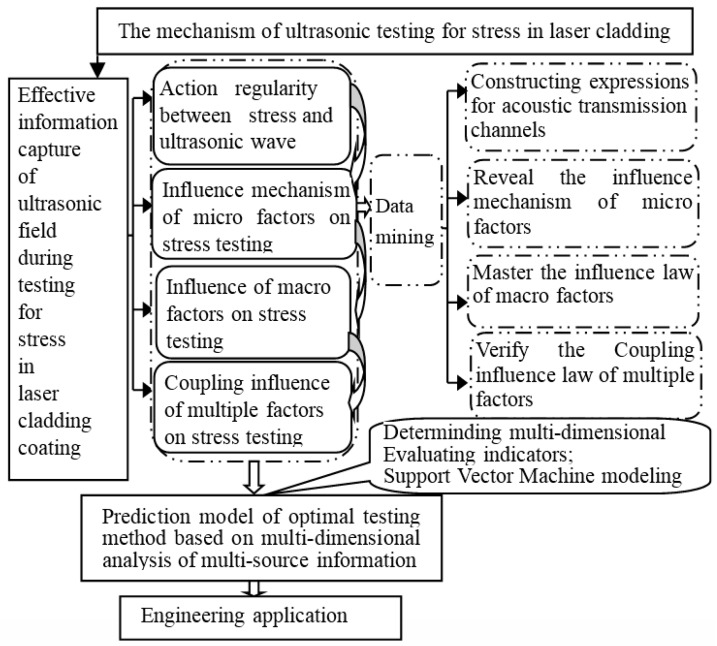
Technology roadmap.

A comprehensive technology roadmap for ultrasonic testing for stress in remanufacturing laser cladding coating is presented based on the above analysis. Figure 10 is used to illustrate the specific technical route process.

## 5. Conclusions and Future Outlook

Laser cladding, due to its technological advantages, has become an important method of green remanufacturing for old products. However, practice shows that stress in laser cladding coating is one of the key factors that affects the service performance and life cycle of remanufacturing parts. In order to realize nondestructive, fast, and reliable testing for the stress in remanufacturing laser cladding coating, ultrasonic nondestructive testing technology was adopted. This paper discusses the active mechanism of micro/macro factors to achieve the reliability of stress measurement and the impact of stress measurement on the quality and safety of remanufacturing components. Strategies to obtain nondestructive, rapid, and accurate measurements of stress in remanufacturing laser cladding coating are also discussed in this paper. Based on the review, the most promising approaches to solve the main issues related to this topic are as follows:

(1) Master the influence law and mechanism of stress in laser cladding coating on ultrasonic testing signals, which is the basis and key to evaluating the stress in laser cladding coating. The transient dynamics and viscoelastic absorbing boundary techniques [78,79] can be introduced into the finite element analysis technique for ultrasonic testing for stress in laser cladding coating. The full-effective ultrasonic field information in testing for stress in laser cladding coating can be collected through finite element postprocessing. The relationship between stress and characteristic parameters of ultrasonic signals can be refined by using data mining technology, so as to determine the influence law and mechanism of stress in laser cladding coating on the ultrasonic signal.

(2) Clarify the mechanisms of texture, inclusion, defect, and other micro factors affecting the result of ultrasonic testing for stress in laser cladding coating, and weaken or separate its influence effectively, which is the key to improving the reliability of ultrasonic testing for stress. The theoretical model of ultrasonic propagation is established by using the semi-analytical method. The full-effective ultrasonic field information in testing for stress in laser cladding coating can be obtained through numerical simulation. The stress testing methods of “insulation” and “sensitivity” to tissue effect are selected by cluster analysis [80] and threshold setting; the corresponding methods are further extended to the experimental observation stage. Combined with elastic-plastic deformation and nonlinear dynamics theory, the essential principle of separating or weakening the tissue effect can be clarified.

(3) Building a prediction model of the optimum testing method based on multidimensional analysis and multi-source information is the key to realizing nondestructive, fast, and reliable evaluations of stress in laser cladding coating under multi-factor coupling actions. Based on the results of multi-factor coupling experimentation, the kernel, principal component analysis (KPCA) [81] is used to extract the multi-dimensional indexes (stress sensitivity, separation organization effect, detection signal recognition, and probe layout convenience) that affect the testing effect. The digital representation method of each dimension index is defined, and its function to indicate the feasibility of the detection method is inversed. The support vector machine method is used to establish the prediction model of the optimum testing method.
